# Aortic Dissection After TAVR: Mechanisms, Management, and Outcomes From a Single-Center Experience

**DOI:** 10.1016/j.jaccas.2026.109331

**Published:** 2026-07-15

**Authors:** Kyle W. Prochno, Maria G. Lopez-Trevino, Fernando Ramirez-Del Val, Kristina Montez, Marcell Szekely, Michael Reardon, Sachin S. Goel, Joe Aoun, Neal S. Kleiman, Marvin Atkins

**Affiliations:** a Houston Methodist DeBakey Heart & Vascular Center, Houston Methodist Hospital, Houston, Texas, USA; b Texas A&M University School of Engineering Medicine, Houston, Texas, USA; c Department of Cardiovascular Surgery, Central Hospital of Northern Pest, Budapest, Hungary; d Department of Cardiology, Houston Methodist Hospital, Houston, Texas, USA

**Keywords:** aortic dissection, endovascular therapy, (TAVR) transcatheter aortic valve replacement

## Abstract

**Background:**

Aortic dissection is a rare but catastrophic complication of transcatheter aortic valve replacement (TAVR), with limited data on mechanisms, management, and outcomes.

**Objectives:**

To describe the incidence, mechanisms, management, and outcomes of aortic dissection complicating TAVR.

**Methods:**

We retrospectively analyzed patients developing perioperative acute aortic dissection during TAVR between 2014 and September 2025. The primary outcome was in-hospital mortality.

**Results:**

Among 2,646 TAVR procedures, 10 patients (0.38%) developed acute aortic dissection. Mean age was 83 ± 8 years, 70% were female, and mean Society of Thoracic Surgeons risk of mortality was 3.6% ± 3.4%. Six dissections (60%) were Stanford type A and 4 (40%) type B. Suspected mechanisms included delivery system manipulation (60%) and wire/catheter trauma (40%). Management consisted of open surgery (30%), endovascular/hybrid intervention (40%), or conservative treatment (30%). In-hospital and 30-day mortality were 40%.

**Conclusions:**

Aortic dissection after TAVR is rare but associated with substantial early mortality, requiring prompt recognition and individualized multidisciplinary management.

## Visual Summary

**Figure:**
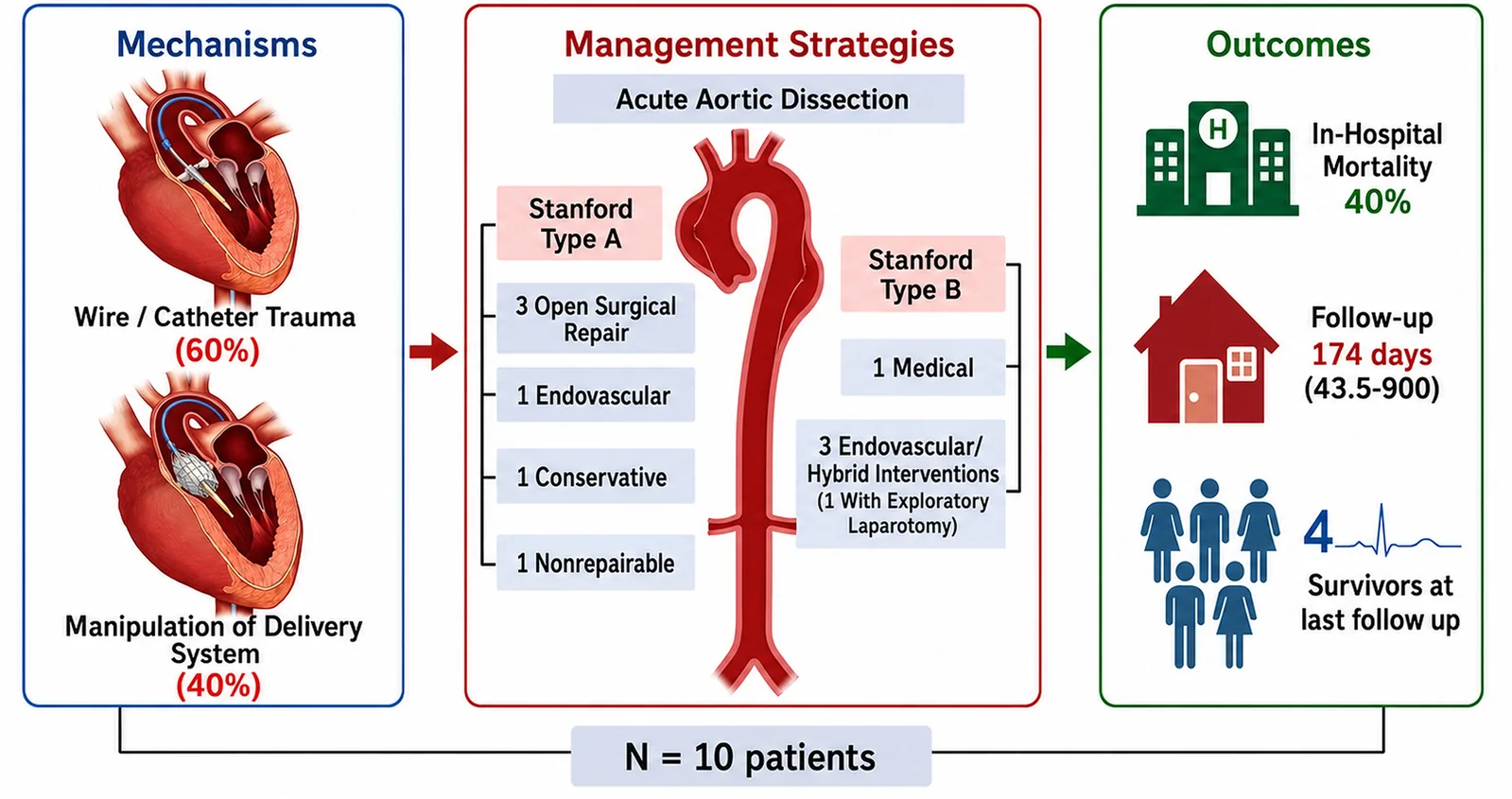

**Visual Summary:**
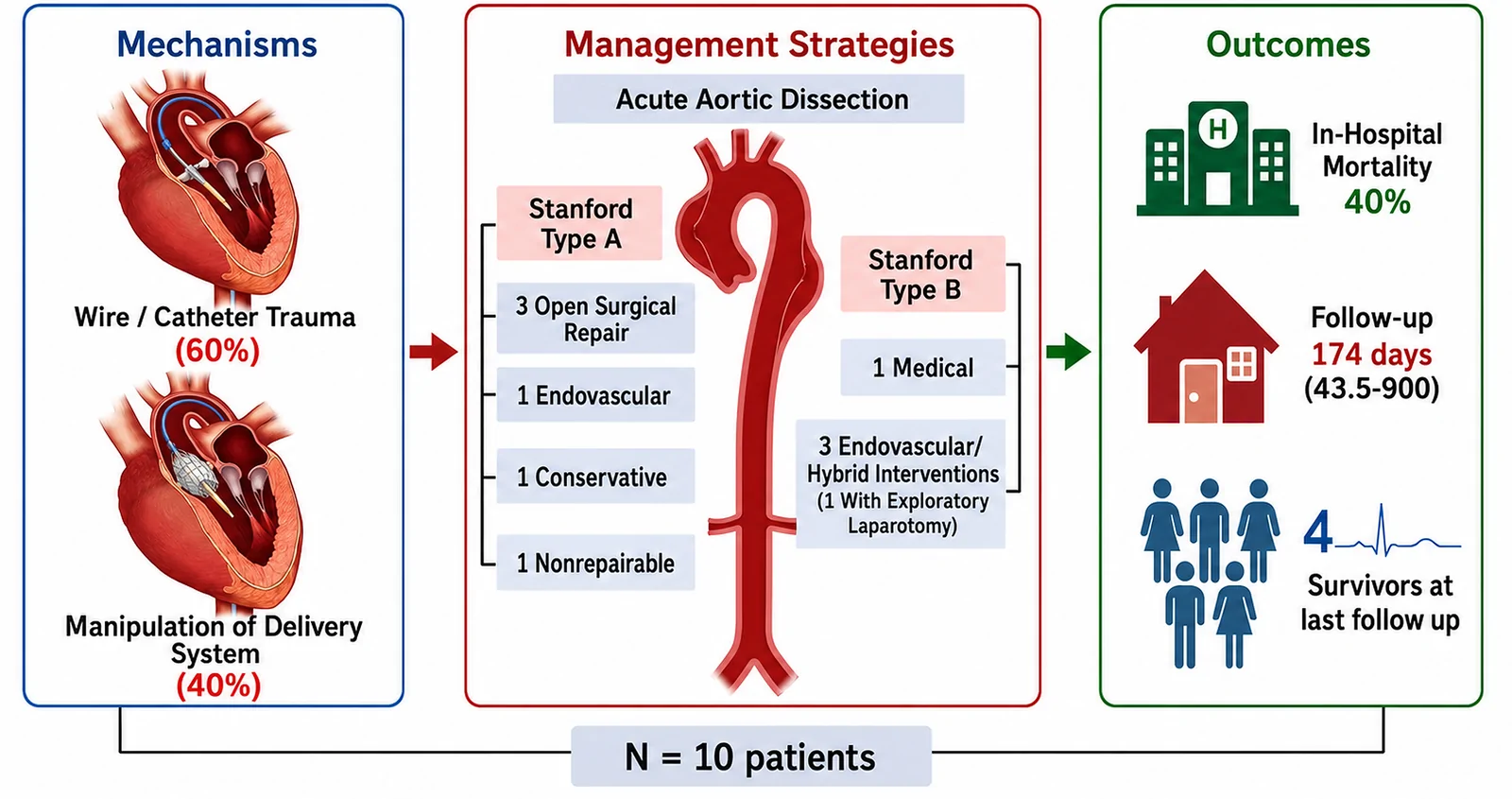
Suspected Mechanisms of Injury, Classification of Dissection, Management Strategies, and Clinical Outcomes Observed in This 10-Year Single-Center Experience TAVR = transcatheter aortic valve replacement.

Transcatheter aortic valve replacement (TAVR) has become an established therapy for severe symptomatic aortic stenosis across surgical risk strata.^1^ As TAVR use has expanded, increasing attention has focused on rare but life-threatening structural complications. Among the most devastating are injuries to the aortic root, ascending aorta, and thoracic or abdominal aorta, which may occur from mechanical trauma related to stiff guidewires, catheters, delivery systems, balloon valvuloplasty, or manipulation within a heavily calcified or fragile aorta.^2^^,^^3^

TAVR-associated aortic dissection remains uncommon, with reported incidences of approximately 0.1% to 0.5%, but it carries substantial morbidity and mortality.^4^, ^5^, ^6^ Existing evidence is largely limited to case reports and small series, and therefore the mechanisms, presentation, management strategies, and outcomes remain incompletely characterized.

We describe a single-center clinical case series of 10 patients who developed acute aortic dissection during or after TAVR over a 10-year period, with emphasis on procedural mechanisms, dissection patterns, rescue strategies, and clinical outcomes.

## Methods

We performed a retrospective, single-center review of all patients undergoing TAVR at a tertiary referral center with an established transcatheter valve and aortic surgery program. The study was approved by the institutional review board, which waived the requirement for individual informed consent because of the retrospective design and minimal risk.

Patients were identified from the institutional TAVR database between 2014 and September 2025. Acute aortic dissection was defined as a new intimal flap detected by intraprocedural aortography, transesophageal echocardiography, computed tomography, or other perioperative imaging. Baseline characteristics, preprocedural computed tomography measurements, procedural variables, imaging findings, management strategies, and outcomes were abstracted from the electronic medical record and procedural reports.

Dissections were classified as Stanford type A or type B based on anatomic extent. Suspected mechanism was determined from procedural documentation and imaging review and categorized as delivery system manipulation or wire/catheter trauma. Management strategies were categorized as open surgical repair, endovascular or hybrid intervention, conservative management, or nonrepairable injury. The primary outcome was in-hospital mortality. Continuous variables are reported as mean ± SD or median with IQR, and categorical variables as counts and percentages.

## Results

### Incidence and baseline characteristics

Among 2,646 TAVR procedures performed during the study period, 10 patients developed acute aortic dissection, corresponding to an incidence of 0.38%. Median age was 83.5 years (IQR: 81.5-89.0), and 7 patients were female. Mean Society of Thoracic Surgeons predicted risk of mortality was 3.6% ± 3.4%.

The cohort had a high burden of comorbidities. Hypertension was present in 8 patients; congestive heart failure and hyperlipidemia in 6 each; prior malignancy and atrial fibrillation in 5 each; and chronic kidney disease, coronary artery disease, and mitral valve disease in 4 each. One patient had undergone prior TAVR with suspected hypo attenuated leaflet thickening, one had prior surgical aortic valve replacement with root enlargement and was noted to have situs inversus and another had prior coronary artery bypass grafting.

Preprocedural computed tomography demonstrated a mean aortic valve calcium score of 2,449 ± 1,244 Hounsfield units. Mean aortic root diameter was 32.9 ± 4.9 mm, mean maximum annular diameter was 24.6 ± 6.1 mm, and mean maximum ascending aortic diameter measured 40 mm above the annulus was 34.1 ± 5.1 mm. Baseline and anatomic characteristics of the cohort are summarized in Table 1.

**Table 1:** Baseline and Anatomic Characteristics of Patients With Transcatheter Aortic Valve Replacement–Associated Aortic Dissection (N = 10)

Age, y	83.5 (81.5-89.0)
Female sex	7 (70)
Former smoker	4 (40)
Valve type – Medtronic Evolut family	9 (90)
Valve type – Edwards Sapien 3 Ultra	1 (10)
Aortic valve calcium score^a^	2,128 (1,472-3,577)
Aortic root diameter, mm^a^	32 (30.5-36.25)
Annulus diameter, mm^a^	25.5 (19.25-27.75)
Maximum ascending aortic diameter at 40 mm above annulus, mm^a^	35 (30-38)
Mean Society of Thoracic Surgeons predicted risk of mortality, %	3.6 ± 3.4

Values are n (%) or median (IQR) unless otherwise indicated.

a Calculated among patients with available data.

### Procedural characteristics and diagnosis

All procedures except 1 were performed via transfemoral access; 1 was performed via transaxillary access. Self-expanding valves were used in 9 patients and a balloon-expandable valve in 1. Predilation was performed in 2 patients and postdilation in 3.

Aortic dissection was identified intraoperatively in 7 patients. These cases were typically recognized after new or worsening paravalvular leak, new pericardial effusion, hemodynamic instability, or abnormal transesophageal echocardiographic findings raised suspicion. Diagnosis was established by visualization of an intimal flap on transesophageal echocardiography and/or confirmation with intraoperative aortography. Three dissections were identified after completion of the procedure, including 2 in the early postoperative period and 1 on postoperative day 1. These patients presented with chest pain, hemodynamic instability, hypotension, or signs of malperfusion.

### Dissection pattern and mechanism

Six dissections were Stanford type A and 4 were Stanford type B. The suspected mechanism was delivery system manipulation in 6 patients and wire or catheter trauma in 4. All type A dissections were attributed to confirmed or suspected delivery system manipulation, whereas all type B dissections were attributed to wire or catheter trauma.

Among Stanford type A dissections, 2 demonstrated retrograde extension into the abdominal aorta, 1 remained confined to the ascending aorta without propagation, and 3 progressed in an antegrade fashion. Among the 2 patients with postoperative Stanford type B dissection who subsequently died, blood pressure after TAVR was 130/59 mm Hg and 95/46 mm Hg, respectively.

**Figure 1:**
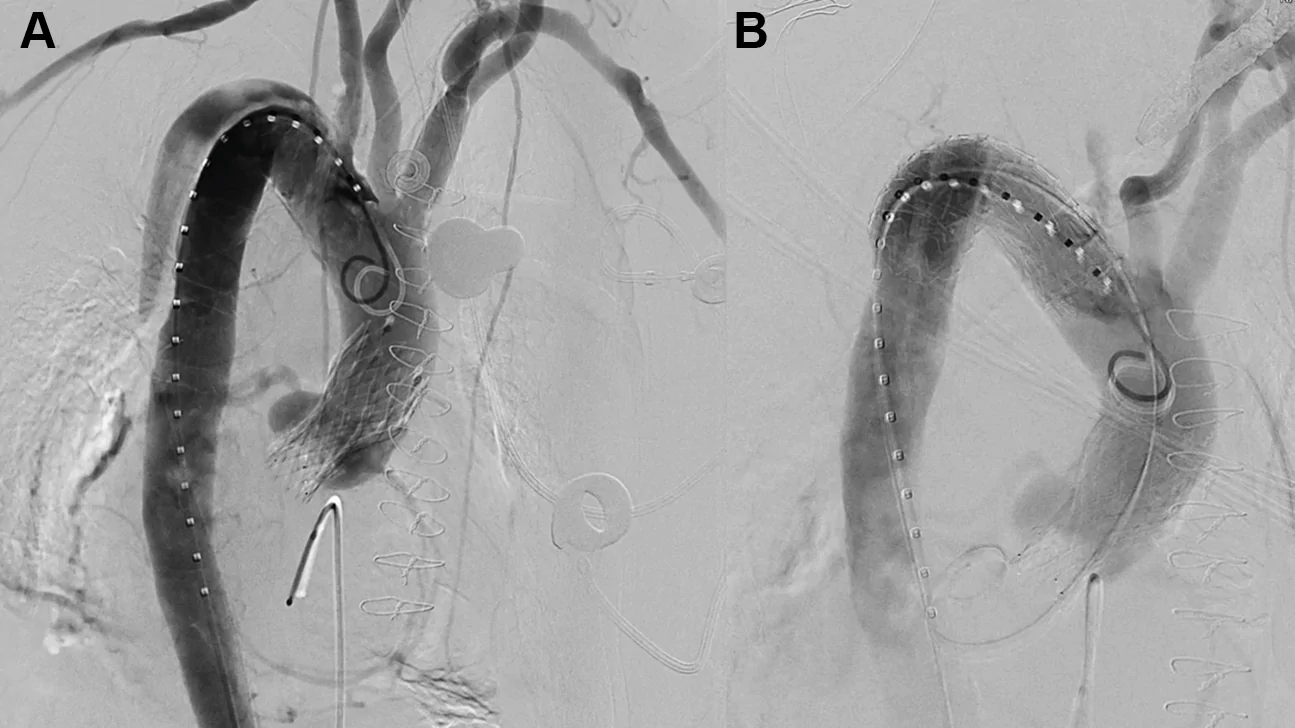
Acute Stanford Type B Aortic Dissection With TEVAR Repair in Situs Inversus Totalis Representative angiographic imaging demonstrating (A) acute Stanford type B aortic dissection extending proximally to the right subclavian artery and (B) successful thoracic endovascular aortic repair (TEVAR) of the dissection in a patient with situs inversus totalis following transcatheter aortic valve replacement.

**Figure 2:**
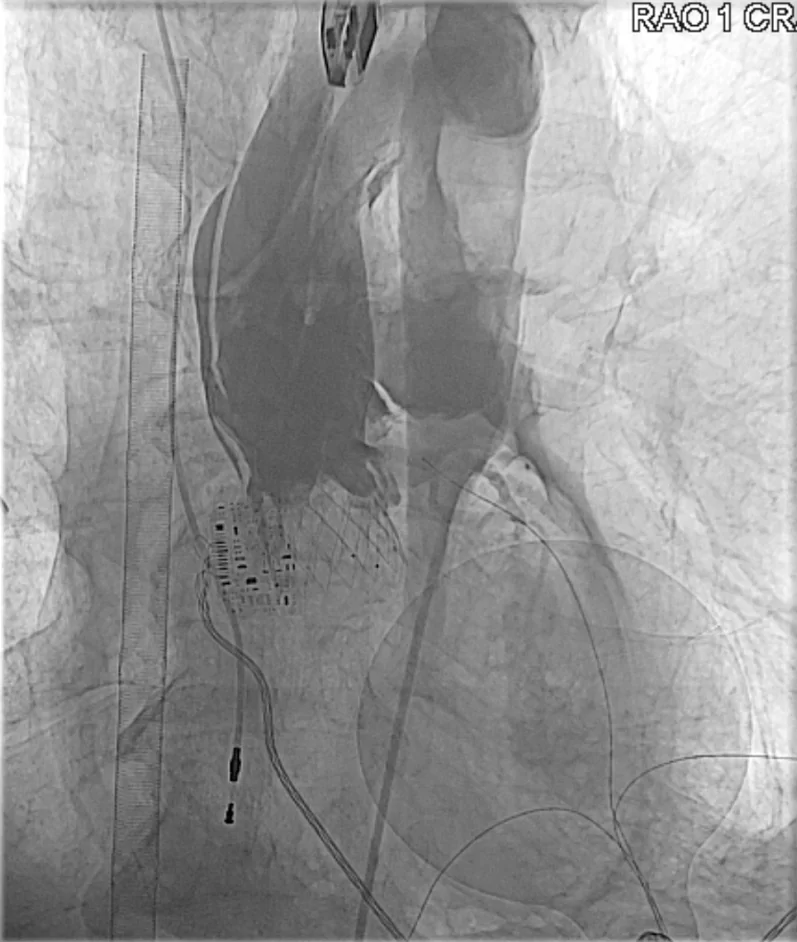
Acute Stanford Type A Aortic Dissection With Aortic Rupture Fluoroscopic angiography demonstrating acute Stanford type A aortic dissection with contrast opacification of the false lumen and rupture into the left hemithorax.

Representative angiographic examples of Stanford type B aortic dissection in a patient with situs inversus totalis and Stanford type A aortic dissection with rupture are shown in Figures 1 and 2, respectively.

### Management strategies

Management varied according to dissection type, anatomic extent, hemodynamic status, and patient-specific operative risk.

Of the 6 patients with Stanford type A dissection, 3 underwent open surgical repair. Procedures included surgical aortic valve replacement with hemiarch reconstruction in 2 patients and root replacement with hemiarch repair in 1. One patient underwent endovascular ascending aortic stent grafting; representative angiographic imaging demonstrates a dynamic ascending aortic intimal flap, extension into the abdominal aorta, and successful exclusion of the false lumen after endovascular ascending stent graft repair (Video 1). One patient with a small, nonpropagating ascending aortic dissection just above the transcatheter valve frame was managed conservatively with intraoperative observation because the dissection remained stable without propagation over 30 minutes. One patient developed cardiac arrest from a ruptured dissection extending into the arch toward the subclavian artery; cardiopulmonary bypass flow could not be established, and the lesion was deemed nonrepairable.

Of the 4 patients with Stanford type B dissection, 1 with a stable descending thoracic dissection was managed medically with close monitoring in the cardiovascular intensive care unit. Three underwent endovascular or hybrid intervention. One patient with right subclavian artery extravasation underwent self-expanding covered stent graft placement. One patient with descending thoracic aortic dissection underwent thoracic endovascular aortic repair after intravascular ultrasound demonstrated proximal propagation toward the arch, raising concern for retrograde type A extension. One patient with abdominal aortic dissection and rupture underwent emergency abdominal aortic stent graft placement with exploratory laparotomy (Video 2). Patient-level characteristics, management strategies, and outcomes are summarized in Table 2, whereas valve type and aortic root anatomy are detailed in Table 3.

**Table 2:** Characteristics, Management, and Outcomes of TAVR-Associated Aortic Dissection

Case #	Age, y	Sex	Dissection Type	Suspected Mechanism	Rescue Strategy	Timing of Identification	In-Hospital Mortality
1	92	M	Stanford Type B	Wire or catheter trauma	Conservative/observation	Postoperative day 1	No
2	82	F	Stanford Type B	Wire or catheter trauma	Hybrid: endovascular abdominal aortic stent grafting + exploratory laparotomy	Immediate postoperative	Yes
3	86	M	Stanford Type A	Delivery system manipulation	Endovascular intervention (ascending aortic stent grafting)	Intraoperative	No
4	85	F	Stanford Type B	Wire or catheter trauma	Endovascular intervention (covered stent grafting of right subclavian artery)	Intraoperative	Yes
5	68	F	Stanford Type A	Delivery system manipulation	Open surgery (SAVR + hemiarch)	Intraoperative	No
6	81	F	Stanford Type A	Delivery system manipulation	Open surgery (ascending aortic replacement)	Intraoperative	Yes
7	81	M	Stanford Type A	Delivery system manipulation	Nonrepairable aortic injury	Intraoperative	Yes
8	72	F	Stanford Type A	Delivery system manipulation	Open surgery (SAVR + hemiarch)	Intraoperative	No
9	91	F	Stanford Type A	Delivery system manipulation	Conservative/observation	Intraoperative	No
10	87	F	Stanford Type B	Wire or catheter trauma	Endovascular intervention (TEVAR)	Immediate postoperative	No

SAVR = surgical aortic valve replacement; TAVR = transcatheter aortic valve replacement; TEVAR = thoracic endovascular aortic repair.

**Table 3:** Valve Type and Aortic Root Anatomy of Patients With TAVR-Associated Aortic Dissection

	Valve Type	AV-CS	Aortic Root (mm)	Annulus Diameter (mm)	SOV –L (mm)	SOV-NC (mm)	SOV-R (mm)	ASC AO at 40 mm (mm)	Notes
1	Evolut R 34 mm	3,972	37	29	39	37	41	41	
2	Evolut Pro Plus 23 mm	—	32	16	—	—	—	37	
3	Evolut Pro Plus 34 mm	3,616	42	36	38	39	36	38	
4	Evolut FX 29 mm	1,200	30	26	28	27	26	30	
5	Evolut FX 34 mm	3,538	32	28	35	35	32	39	
6	Evolut FX 26 mm	1744	28	25	26	26	24	27	
7	Evolut FX 29 mm	2,128	37	27	35	35	34	33	
8	Evolut FX 26 mm	946	34	23	31	31	30	38	
9	Edwards Sapien 3 Ultra 20 mm	—	25	18	—	—	—	28	Previous TAVR, with likely HALT
10	Evolut FX 23 mm	—	32	18	—	—	—	30	Previous SAVR with root enlargement. Situs inversus totalis

Values are presented for patients with available data.

— = data not available; ASC AO = ascending aorta; AV-CS = aortic valve calcium score; NC = noncoronary; SOV = sinus of valsalva; other abbreviations as in Table 2.

### Outcomes

Both in-hospital and 30-day mortality were 40% (4 of 10). Two patients died intraoperatively: 1 with a nonrepairable ruptured Stanford type A dissection and 1 with Stanford type B dissection complicated by abdominal aortic rupture requiring emergency stent graft placement and exploratory laparotomy.

Two additional patients died during the index hospitalization. One death occurred in a patient with Stanford type B dissection who developed hemorrhagic shock, severe coagulopathy, and multiorgan failure in the immediate postoperative period after self-expanding covered stent graft placement for right subclavian artery injury. The other occurred in a patient with Stanford type A dissection who developed cardiogenic shock due to irreversible biventricular failure 2 days after open repair for ascending aortic perforation.

Among the 6 patients who survived hospital discharge, median follow-up was 174 days (IQR: 43.5-900 days). Two patients died after discharge at 42 days and 2,126 days, respectively. The later death was attributed to pulmonary embolism and was not considered aortic-related; the cause of death for the patient who died 42 days after discharge was not available. At last follow-up, 4 patients were alive and free from aortic-related reintervention.

## Discussion

In this single-center clinical case series, acute aortic dissection after TAVR was rare, occurring in 0.38% of procedures, but was associated with high early mortality. Most dissections were Stanford type A, most were recognized intraoperatively, and suspected mechanisms were predominantly related to delivery system manipulation or wire/catheter trauma. Management strategies were heterogeneous and included open repair, endovascular or hybrid intervention, conservative observation, and nonrepairable injury.

Our findings are consistent with prior reports describing TAVR-associated aortic dissection as a low-frequency but high-impact complication.^4^, ^5^, ^6^ The observed incidence falls within the range previously reported, whereas the 40% 30-day mortality underscores the catastrophic nature of this event. Although the small sample size limits comparison across strategies, survival in selected patients appeared possible when diagnosis was rapid and definitive rescue therapy was immediately available.

Several clinically relevant observations emerged. First, dissection mechanism appeared to differ by anatomic pattern. All type A dissections were associated with delivery system manipulation, whereas type B dissections were associated with wire or catheter trauma. This distinction highlights the need for careful manipulation of stiff wires and delivery systems, particularly in patients with calcified, tortuous, or fragile aortas.^2^^,^^3^

Second, 70% of affected patients were female. Although this series was not designed to identify predictors of dissection and lacks a control group, this observation may be relevant because female patients often have smaller annular and aortic dimensions. Oversizing was not identified as a primary mechanism in our cohort; however, smaller vascular anatomy may plausibly increase vulnerability to injury during device delivery, valve positioning, or balloon dilation. This finding should be interpreted cautiously and requires further study.

Third, type A dissection after TAVR did not result in a uniform management strategy. Three patients underwent open repair, 1 underwent endovascular ascending aortic stent grafting, 1 was managed conservatively because the dissection was limited and nonpropagating, and 1 injury was nonrepairable. This reflects the individualized decision-making required in this frail, high-risk population. Although urgent open repair remains the standard approach for most patients with type A dissection who are acceptable surgical candidates, selected cases may require endovascular or conservative strategies based on anatomy, stability, and operative risk.^7^, ^8^, ^9^, ^10^

The role of the multidisciplinary heart team was central in this series. Management of TAVR-associated aortic dissection requires immediate recognition, rapid imaging interpretation, hemodynamic stabilization, and access to cardiac surgery and endovascular expertise. In our center, interventional cardiologists, imaging cardiologists, cardiac surgeons, anesthesiologists, and endovascular specialists were involved in procedural planning and rescue management. This integrated model enabled rapid escalation to open, endovascular, or hybrid intervention when appropriate.

### Study limitations

This study is limited by its retrospective design, small sample size, and single-center setting. Because all cases occurred at a tertiary center with immediate access to experienced aortic surgeons and endovascular expertise, findings may not be generalizable to all TAVR programs. Mechanisms of injury were determined from procedural reports and imaging review and may be subject to misclassification. The study was descriptive and not designed to identify predictors of dissection or compare outcomes across management strategies.

## Conclusions

Aortic dissection after TAVR is rare but frequently catastrophic. In this 10-patient clinical case series, dissections were most often related to delivery system manipulation or wire/catheter trauma, and management required individualized open, endovascular, hybrid, or conservative strategies. Early recognition, immediate imaging, and coordinated multidisciplinary rescue management are essential to optimize outcomes.

Perspectives **COMPETENCY IN MEDICAL KNOWLEDGE:** Aortic dissection is a rare but life-threatening complication of TAVR associated with high early mortality and heterogeneous management strategies.

**COMPETENCY IN PATIENT CARE:** Early recognition using intraoperative imaging and prompt escalation to surgical or endovascular intervention are critical to improving outcomes.

**TRANSLATIONAL OUTLOOK:** Future studies are needed to define procedural risk factors and develop standardized management algorithms for TAVR-associated aortic dissection.

## Funding Support and Author Disclosures

This study received no external funding. Dr Reardon is a consultant for Medtronic, Boston Scientific, Abbott, and W. L. Gore & Associates. Dr Kleiman is a local principal investigator for trials sponsored by Boston Scientific, Medtronic, Abbott, and Edwards Lifesciences. Dr Goel is a consultant for Medtronic, JC Medical, Boston Scientific, and W. L. Gore & Associates; and serves on the Speakers Bureau for Abbott Structural Heart. All other authors have reported that they have no relationships relevant to the contents of this paper to disclose.

**Take-Home Messages**

• TAVR-associated aortic dissection does not mandate a single treatment strategy; management should be tailored according to dissection extent, hemodynamic status, and patient-specific operative risk.
• Selected patients may be successfully managed with surgical, endovascular, hybrid, or even conservative approaches when guided by anatomy and clinical presentation.

## Appendix

Video 1 Representative Angiographic Imaging From Patient 3 Demonstrating Acute Stanford Type A Aortic Dissection During TAVR The video shows a dynamic intimal flap in the ascending aorta, extension of the dissection into the abdominal aorta, and successful treatment with endovascular ascending aortic stent grafting.

Video 2 Representative Fluoroscopic Angiography Demonstrating Acute Stanford Type B Aortic Dissection With Abdominal Aortic Rupture Following TAVR The video illustrates endovascular abdominal aortic stent graft deployment performed as emergency rescue therapy.
